# COVID-19 Vaccine Acceptance and Its Determinants in the General Population of Delhi, India: A State Level Cross-Sectional Survey

**DOI:** 10.7759/cureus.26936

**Published:** 2022-07-17

**Authors:** Pragya Sharma, Saurav Basu, Suruchi Mishra, Nutan Mundeja, B S Charan, Gautam Singh, Mongjam M Singh

**Affiliations:** 1 Community Medicine, Maulana Azad Medical College, New Delhi, IND; 2 Indian Institute of Public Health-Delhi, Public Health Foundation of India, New Delhi, IND; 3 Public Health, Government of National Capital Territory, New Delhi, IND

**Keywords:** vaccine coverage, vaccine acceptance, vaccine hesitancy, covid-19, sars-cov-2

## Abstract

Objective: To ascertain the COVID-19 vaccination acceptance and the factors contributing to vaccine hesitancy and vaccine confidence in the adult population, and the intention for vaccination of their children.

Methods: This cross-sectional analysis reports the ancillary results of a population-based SARS-CoV-2 serosurvey conducted in Delhi, India, from September 24 to October 14, 2021. Data were collected from 20312 adult participants through a multistage sampling method from all the 274 wards in the 11 districts of the national capital territory region.

Results: We enrolled 12093 (59.3%) females and 8219 (40.5%) male participants with mean (SD) age of 40.3 (14.6) years. The vaccine acceptance rate in the participants was 67.7% (95% CI 67.1, 68.4), with 6031 (43.8%) having received one dose and 7727 (56.2%) having received two vaccine doses. On adjusted analysis, lack of vaccine acceptance was independently associated with female gender aOR 1.15 (95% CI 1.1, 1.23), younger age-group (18-49 years) aOR 1.85 (95% CI 1.71, 2.0), low educational status aOR 1.88 (95% CI 1.77, 2.0), in those with no history of COVID-19 aOR 1.81 (95% CI 1.69, 1.95), non-healthcare workers aOR 2.1 (95% CI 1.7, 2.53), and in the absence of hypertension comorbidity aOR 1.22 (1.1, 1.38). Lack of awareness of COVID-19 vaccines, including doubts on vaccine efficacy and long-term safety, were primary drivers of vaccine hesitancy in the unvaccinated subgroup. Only 35.6% participants reported a positive intention to vaccinate their children.

Conclusions: One in three adults lacked vaccine acceptance. High prevalence of delay in second dose vaccination was also observed.

## Introduction

The Coronavirus 2019 (COVID-19) pandemic has emerged as the most important public health challenge of the 21^st^ century and has caused enormous health and economic losses globally [1]. Vaccination is considered the most important intervention for inhibiting SARS-CoV-2 transmission and combating the pandemic [2]. Real-world studies globally have indicated the effectiveness of several COVID-19 vaccines in preventing severe disease, hospitalization, and death, especially in the elderly and the immunocompromised [3,4].

Vaccine hesitancy, as per the World Health Organization (WHO), is the refusal or delay in acceptance of vaccination despite the availability, accessibility, and affordability of vaccination services [5]. Vaccine hesitancy is one of the leading threats to global health and needs to be sufficiently addressed to safeguard public health, especially during the COVID-19 pandemic [6].

Population-based studies from several countries reported the willingness to receive these vaccines in adults was variable, ranging from 28.4% to 97%, but the median willingness was higher in low-income compared to higher and middle-income countries [6-8]. The level of vaccine hesitancy is influenced by several factors, such as age, gender, perceived susceptibility to COVID-19 infection, and the perceived efficacy and safety of the available vaccines [6,9]. Moreover, the intention to vaccinate children may be lacking in nearly one in five parents and caregivers [10].

India with 138 million population till 31^st^ October 2021 recorded 3,42,73,300 COVID-19 cases and 4,58,186 deaths attributed to the disease [11]. The country experienced a major second wave of the COVID-19 pandemic from April to June 2021, with high morbidity and mortality predominantly caused by the circulation of the SARS-CoV-2 Delta variant having extremely high transmissibility and immune escape mechanisms [12,13]. The lack of vaccination is nearly 85%-90% of the adult population was a major factor contributing to the high rate of hospitalization and death during the second wave of the COVID-19 pandemic in India [13].

In January 2021, the Indian regulatory agency provided emergency use authorization for ChAdOx1 nCoV-19 (Covishield, Serum Institute of India, Pune), India manufactured version of the Oxford AstraZeneca (AZD222) vaccine. It also provided EUA in clinical trial mode for BBV152 (Covaxin; Bharat Biotech International, Hyderabad). The COVID-19 vaccination campaign was launched on January 16, 2021, for health and frontline workers in the first stage. In the second stage, it was expanded to the elderly (>60 years old) and comorbid (>45 years old) individuals (March 1, 2021, onwards), third stage to all >45 years old (April 1, 2021, onwards), and fourth stage all >18 years old (May 1, 2021, onwards), and fifth stage 15-18 years old (January 3, 2022, onwards). Vaccination was provided free of cost to all eligible residents conveniently in designated facilities of their choice by a health team with private health facilities having a very minor role. Registration for vaccination was done through CoWIN, the Indian government web portal for the management of COVID-19 vaccination registration and generation of vaccination certificates. Walk-in vaccination with on-site registration was also made available to enable those without internet or mobile services to avail of these vaccination services [14]. As of 21^st^ October 2021, 19.8 million cumulative vaccine doses were administered to the eligible beneficiaries of the state, while as of 11^th^ July 2022, 35.2 million doses were provided, including 2.84 million doses in the under 18 population [11].

Globally, the disparity in vaccination has emerged as a major threat in halting the COVID-19 pandemic, with only 8.3% of the population in low-income countries having received at least one dose of a COVID-19 vaccine in 2021 [15]. Although limited vaccine availability contributes significantly to lower vaccination coverage in the developing world, the experience of countries such as the USA [16] and India [17] suggests the role of vaccine hesitancy in lowering vaccine acceptance despite sufficient vaccine stocks.

We conducted this study with the objective of ascertaining the COVID-19 vaccine acceptance and the factors contributing to vaccine hesitancy and vaccine confidence in the adult population. We also estimated the intention for vaccination of children in the participants. The study findings can guide policymakers in addressing factors that drive COVID-19 vaccine hesitancy and achieve and plan for the accelerated expansion of vaccination coverage in similar settings. 

## Materials and methods

This cross-sectional analysis reports the ancillary results of a population-based SARS-CoV-2 serosurvey conducted in Delhi from September 24 to October 14, 2021 [18], a period nearly two and a half months after the end of the second wave of the COVID-19 pandemic in Delhi. 

The participants included adult residents of Delhi selected from all the 274 wards of Delhi, the national capital territory with ~19 million population. Within each ward, a multi-stage sampling method was applied for the selection of the participants. First, the designated medical officer at every urban primary health center obtained a line list of the major settlements in their ward that were classified as either planned colonies, urban slums, resettlement colonies, unauthorized colonies, or rural areas. From each ward, the number of participants to be selected per settlement type was determined as proportional to the population size of the latter. The survey areas/localities from each type of settlement available in each ward were selected from the line list using the simple random sampling method. Next, from each selected survey area, individual households were further selected through the systematic random sampling method. Finally, within each selected household, a single participant was selected using the simple random sampling method after enlisting all members of the household in ascending order of their ages, a process known as the age-order procedure. 

The primary outcome of this analysis was vaccine acceptance which was considered to correlate with actual vaccine intake. The estimated sample size was adequate at a 95% confidence level, 60% expected vaccine confidence, 1% absolute precision, and a design effect of two. 

Data were collected electronically using a customized android tablet application by field personnel accompanied by the local frontline health workers. The following information was collected: (1). COVID-19 vaccination status (2). The number of doses of vaccine received and the date(s) of vaccination (3). Sociodemographic characteristics (age, gender, education, occupation) (4). History of COVID-19 infection with laboratory confirmation (5). Comorbidities: Diabetes mellitus and hypertension. Furthermore, in the unvaccinated, reasons for the absence of vaccination were queried through close-ended statements, which were assessed for face validity by a group of experts. The thematic domains for non-vaccination were formulated based on a conceptual framework related to the health belief model, including lack of vaccine-related awareness, perceived fear of side effects, perceived lack of safety, perceived lack of susceptibility, and perceived barriers (administrative, operational, and familial) [19]. 

The data were analyzed using IBM Corp. Released 2017. IBM SPSS Statistics for Windows, Version 25.0. Armonk, NY: IBM Corp. Summary statistics of socio-demographic, clinical, and vaccination parameters of the participants were reported. On bivariate analysis, the association between participant characteristics and vaccination status was assessed using the chi-square test. The explanatory variables that were found to have a statistically significant association with lack of vaccine acceptance were included in a binary logistic regression model. A p<0.05 was considered statistically significant.

The study was approved by the Institutional Ethics Committee, Maulana Azad Medical College & Associated Hospitals, New Delhi, vide F.1/IEC/MAMC/85/03/2021/No 428 dated 21.08.2021. Electronic and informed consent was obtained from all the study participants.

## Results

Sociodemographic characteristics

Data of 20312 adult participants were analyzed by us, including 12093 (59.5%) females and 8219 (40.5%) males. The mean (SD) of the participants was 40.3 (14.6) years. Education status of the participants was up-to primary 6322 (31.2%), middle school 2983 (14.7%), high school 4243 (20.9%), senior secondary 2529 (12.5%), and graduate and above 4226 (20.8%). Occupational status of the participants was unemployed 1086 (5.3%), housewife 8540 (42%), student 1399 (6.9%), daily wager 1458 (7.2%), salaried 4725 (23.3%), individual business 2098 (10.3%), and healthcare workers 778 (3.8%). The residential settlement type of the participants was planned colony 5598 (27.6%), resettlement colony 2173 (10.7%), urban slum 8886 (43.7%), unauthorized colony 1018 (5.0%), and village 2628 (12.9%).

Vaccination status

The vaccine acceptance rate in the participants was 67.7% (95% CI 67.1, 68.4), with 6031 (43.8%) having received one dose and 7727 (56.2%) having received two vaccine doses signifying partial and full vaccination, respectively. The highest rate of vaccination was observed in the New Delhi district (72.9%) and the least in the South district (54.2%) (Table 1).

**Table 1:** Distribution of COVID-19 vaccination status among participants stratified by district residence

District name	Total	Unvaccinated	One dose	Two doses
East	2020	562 (27.8)	590 (29.2)	868 (43.0)
New Delhi	1363	360 (27.1)	325 (23.8)	669 (49.1)
North East	1419	552 (38.9)	345 (24.3)	522 (36.8)
Northwest	3158	995 (31.5)	992 (31.4)	1171 (37.1)
North	1953	625 (32.0)	603 (30.9)	725 (37.1)
Shahdara	1742	589 (33.8)	524 (30.1)	629 (36.1)
South-East	2040	647 (31.7)	617 (30.2)	776 (38.0)
South-West	2558	720 (28.1)	925 (36.2)	913 (35.7)
South	1740	797 (45.8)	425 (24.4)	518 (29.8)
West	2310	689 (29.8)	685 (29.7)	936 (40.5)
Total	20303	6545 (32.2)	6031 (29.7)	7727 (38.1)

The most common sources of vaccine-related health information were family (58.3%), frontline health workers (40.3%), friends (30.6%), and media (20.8%) (Table 2). 

**Table 2:** Distribution of sources of vaccine related information in the participants

Source	Unvaccinated (N=6554) n (%)	Vaccinated (at-least one dose) (N=13758) n (%)	p-value
Family	3567 (54.4)	8277 (60.1)	<0.001
Friends	1732 (26.4)	4489 (32.6)	<0.001
Employer	457 (7)	1300 (9.4)	<0.001
Frontline health worker	2446 (37.3)	5730 (41.6)	<0.001
Local doctor or nurse	603 (9.2)	2038 (14.8)	<0.001
Religious leader	92 (1.4)	294 (2.1)	<0.001
Political leader	129 (2)	423 (3.1)	<0.001
TV news / Newspaper	1231 (18.8)	2993 (21.7)	<0.001
Social media	237 (3.6)	810 (5.9)	<0.001
YouTube	112 (1.7)	413 (3.0)	<0.001

Factors associated with lack of vaccine acceptance

On adjusted analysis, lack of vaccine acceptance was independently associated with female gender aOR 1.15 (95% CI 1.1, 1.23), younger age-group (18-49 years) aOR 1.85 (95% CI 1.71, 2.0), low educational status aOR 1.88 (95% CI 1.77, 2.0), non-healthcare workers aOR 2.1 (95% CI 1.7, 2.53), living in non-planned colonies aOR 1.41 (95% CI 1.31, 1.51, no history of COVID-19 aOR 1.81 (95% CI 1.69, 1.95), and absence of hypertension comorbidity aOR 1.22 (1.1, 1.38) (Table 3).

**Table 3:** Distribution of factors associated with absence of vaccination in eligible population (n=20365) *Dichotomized as planned versus other colonies for regression analysis

Variable	Total	Unvaccinated	Adjusted odds	p-value
Sex				
Male	8215 (40.5)	2378 (28.8)	1	<0.001
Female	12088 (49.5)	4227 (34.9)	1.15 (1.1, 1.23)	
Age				
18-49	14707 (72.4)	5162 (35.1)	1	<0.001
≥50	5596 (27.6)	1383 (24.7)	1.85 (1.71, 2.0)	
Education				
Below High School	9305 (45.8)	3664 (39.4)	1.88 (1.77, 2.0)	<0.001
≥High School	10998 (54.2)	2881 (26.2)	1	
Occupation				
Healthcare Workers	778 (3.8)	123 (15.8)	1	<0.001
Others	19525 (96.2)	6422 (32.9)	2.1 (1.7, 2.53)	
Settlement type*				
Planned	5598	1452 (25.9)	1	<0.001
Unauthorized	1018	389 (38.2)	1.41 (1.31, 1.51)^*^	
Slum	8886	3115 (35.1)		
Resettlement Village	2173 2628	681 (31.3) 908 (34.6)		
History of COVID-19				
Present	5840 (28.8)	1323 (22.7)	1	<0.001
Absent	14463 (71.2)	5222 (36.1)	1.81 (1.69, 1.95)	
Diabetes Mellitus				
Yes	1255 (6.2)	313 (24.9)	1	0.093
No	19048 (93.8)	6232 (32.7)	1.13 (0.98, 1.31)	
Hypertension				
Yes	1856 (9.1)	469 (25.3)	1	0.001
No	18447 (90.9)	6076 (32.9)	1.22 (1.1, 1.38)	

Interval delay in vaccination with the second dose

A higher proportion of participants belonging to the female gender, younger age group (18-49), lower than high school educational status, lack of history of COVID-19, and those living in non-planned colonies had partial vaccination status due to pending second dose, and this difference was statistically significant (p<0.001) (Table 4). 

**Table 4:** Factors associated with pending second dose of vaccination in eligible population (n= 13758)

Variable	Received Second Dose		
	Yes	No	p-value
Ever tested positive			
Yes	2655(58.8)	1862 (41.2)	<0.001
No	5072(54.9)	4169 (45.1)	
Age			
18-49	4599 (48.2)	4946 (51.8)	<0.001
≥50	3128 (74.2)	1085 (25.8)	
Education			
Below High School	2777 (49.2)	2864 (50.8)	<0.001
≥High School	4950 (61.0)	3167 (39.0)	
Settlement type			
Planned	2745(66.2)	1401 (33.8)	<0.001
Unauthorized	311 (49.4)	318 (50.6)	
Slum	3017 (52.3)	2754 (47.7)	
Resettlement	817 (54.8)	675 (45.2)	
Village	837(48.7)	883 (51.3)	
Gender			
Male	3573 (60.9)	2298 (39.1)	<0.001
Female	4154 (52.7)	3733 (47.3)	

In the participants who took the first dose of Covaxin but not the second dose yet (n=889), the interval gap beyond 30 days was observed in 484 (54.4%) participants. The median (IQR) delay beyond this interval gap was 23 (10.2, 54) days.

In the participants who took the first dose of Covishield but not the second dose yet (n=5142), the interval gap beyond 90 days was observed in 731 (14.2%) participants. The median (IQR) delay beyond this interval gap was 17 (7, 43) days.

Reasons for vaccine hesitancy and vaccine confidence

Lack of awareness of COVID-19 vaccines, including doubts about vaccine efficacy and long-term safety, were primary drivers of vaccine hesitancy in the unvaccinated subgroup (Table 5). In contrast, among those vaccinated (n=13758), the most common reasons for vaccine confidence were the belief that vaccines prevent COVID-19 (40.4%), the vaccine prevented severe disease (14.1%), trust in vaccine safety (21.1%), vaccine role in stopping the pandemic (7.5%), going back to normal life (3.7%), advice from a doctor (3.2%), employer (1.6%), family or friend (1.2%).

**Table 5:** Reasons for lack of vaccination in the unvaccinated participants (n=6554)

Reason (multiple response)	Frequency (%)
Do not know much about vaccines	2256 (34.4)
Do not know how effective they are	2039 (31.1)
Do not know if people with comorbidities can take	249 (3.8)
Do not know how to make choice of vaccine	209 (3.2)
Do not know how long to wait post-recovery	69 (1.1)
Worried about safety of vaccine	594 (9.1)
Do not know the long-term effects of vaccine	482 (7.4)
Nurse may not administer properly	116 (1.8)
Vaccine not transported or stored properly	45 (0.7)
People getting COVID-19 after vaccination	57 (0.9)
Worried about side effects of vaccine	460 (7.0)
Will get fever/headache/body ache	284 (4.3)
Will get serious side effects	112 (1.7)
May die after taking vaccine	91 (1.4)
May be sick for long time	89 (1.4)
May affect ability to have children	16 (0.2)
Do not know the process to get vaccinated	643 (9.8)
Do not know if I need to register	314 (4.8)
Do not know how to register in CoWIN application	173 (2.6)
Do not know how to schedule appointment	142 (2.2)
Do not know what documents are needed	51 (0.8)
Do not know where vaccine is available	67 (1.0)
Do not know about walk-in-vaccinations	33 (0.5)
Do not know I am eligible	31 (0.5)
Do not need to get vaccinated	418 (6.4)
I already had COVID-19	116 (1.8)
I do not think I will get COVID-19	189 (2.9)
I will recover even if I have COVID-19	64 (1)
Infections have reduced a lot	30 (0.5)
I want to wait for some more time	51 (0.8)
Unable to get vaccinated despite some willingness	1051 (16)
No vaccination site near my home	291 (4.4)
Difficult or expensive to travel to vaccination site	165 (2.5)
Not finding appointment slots	410 (6.3)
Too much crowd at vaccination sites	358 (5.5)
My family is opposing	84 (1.3)
Lack of time	158 (2.4)
Expensive	4 (0.1)
No stock of preferred vaccine	23 (0.4)
No stock of any vaccine	28 (0.4)
Advised by medical professional to not get vaccinated	366 (5.6)
Pregnant or recent mother with new-born	122 (1.9)
Uncontrolled Diabetes Mellitus	86 (1.3)
Medical complications	43 (0.7)
Recently had COVID-19	66 (1)

Intention for vaccination in children

A total of 7224 (35.6%, 95% CI 57.6, 59.3) participants reported positive intention to vaccinate their children in the household, 2531 (12.5%) denied intention to vaccinate children, 2644 (13.0) were undecided (n=12399). On adjusted analysis, participants who were themselves vaccinated or those who were health workers by occupation had significantly higher odds of having a positive intention for vaccinating their children (Table 6).

**Table 6:** Distribution of factors associated with positive intention to vaccinate children *7913 responses not collected in absence of a child in the household; ^At-least one dose

Variable	Total (N=12399)^*^	Positive intention	Negative / Undecided	Adjusted odds	p-value
Age					
18-49	9965 (80.4)	5865 (58.9)	4100 (41.1)	1.1 (0.99, 1.2)	0.052
≥50	2434 (19.6)	1359 (55.8)	1075 (44.2)	1	
Gender					
Male	4570 (36.9)	2714 (59.4)	1856 (40.6)	1	0.147
Female	7829 (63.1)	4510 (57.6)	3319 (42.4)	0.94 (0.88, 1)	
Education					
Below High School	6424 (51.8)	3678 (57.3)	2746 (42.7)	0.99 (0.92, 1.1)	0.019
≥High school	5975 (48.2)	3546 (59.3)	2429 (40.7)	1	
Occupation					
Healthcare Worker	218 (1.8)	109 (50)	109 (50)	1.4 (1.1, 1.8)	0.015
Others	12181 (98.2)	7115 (58.4)	5066 (41.6)	1	
Vaccination status					
Vaccinated^^^	5981 (48.2)	4001 (66.9)	1980 (33.1)	1	<0.001
Not vaccinated	6418 (51.8)	3223 (50.2)	3195 (49.8)	2 (1.8, 2.1)	

## Discussion

Vaccine hesitancy contributed to the lower rates of vaccination in Indian adults before the second wave of the COVID-19 pandemic in India. The present study conducted after this second wave observed the lack of vaccine acceptance in nearly one in three eligible adults. In most cases, vaccine hesitancy was the most common reason for missing vaccination, generating concerns over vaccine side effects, long-term vaccine safety, doubts over vaccine efficacy, and the perception of lack of susceptibility to the disease. The rates of vaccine acceptance in this study are lower than that observed in the Chinese general population (67.1%-88.6%) [20].

Our findings suggest that frontline health workers constitute a key source of vaccine-related health information in their service areas, such as urban slums and villages, implying the need to be sensitive and train them towards community mobilization for vaccination purposes. Evidence from low-income countries also indicates that healthcare workers are considered the most trustworthy sources of guidance on COVID-19 vaccination [6].

Furthermore, in this study, the non-vaccinated compared with the vaccinated participants were lacking vaccine-related information irrespective of its source, suggesting the need for enhanced focus on the conduct of awareness generation activities in areas with low vaccination coverage. Although adverse social media coverage and misinformation play a significant role in undermining vaccine confidence, in this study, social media was reported as a minor source of vaccine-related information [21].

Television and print media were reported as major sources of vaccine-related health information by the participants. Certain media reports in the initial months after the launch of India’s vaccination campaign expressed reservations about the usefulness, safety, and efficacy of the available vaccines, therefore possibly contributing to the increase in vaccine hesitancy in the general population [22-24].

Our study findings show that vaccination coverage was lower in women and those with lower educational attainments signifying how existing social inequities and reduced empowerment can accentuate vaccine hesitancy and diminish the utilization of vaccination services. This is in contradiction to the finding in the developed world wherein the male gender was associated with vaccine hesitancy [16], the difference occurring probably due to the correlation of low educational status with the female gender in the present study. Nevertheless, the global evidence also suggests that socioeconomically disadvantaged groups have an increased risk of missing out on COVID-19 vaccination due to comparatively reduced accessibility and higher vaccine hesitancy [7-9].

India initiated vaccination in 15-18-year-old children from January 3, 2022, onwards, which was later extended to children 12 or older. Compared to studies in the developed world, the intention to vaccinate children in the present study was much lower [10], suggesting that a significant proportion of even vaccinated parents may have concerns over vaccinating their children, which needs to be sufficiently addressed through effective interventions. Although this study observed that lower vaccine acceptance in individuals is likely to correlate with a reduced intention to vaccinate their children, as of July 11^th^ 2022, nearly ~90 of eligible children and adolescents above >=12 years of age in Delhi have been vaccinated [11] suggestive of minimal parental vaccine hesitancy. 

According to government data, Delhi achieved the target of nearly universal at-least single dose vaccination coverage for its eligible adult population by November-December 2021 [25]. Understanding the real-world experiences of the city-state in accelerating vaccine coverage and overcoming vaccine hesitancy in the population may have important lessons globally. Government initiates last-mile delivery through the ‘Har Ghar Dastak’ (reach every house) campaign involving strict directives for conducting house-to-house surveys, counseling, mobilization, and vaccination of all missed out and eligible beneficiaries by frontline health worker teams. Due list of eligible second dose beneficiaries was also extracted from the CoWIN portal for further tracking [26, 27].

The strengths of this study are that it was conducted in real-world settings with a large sample size which was representative of the diverse population composition in an urban metropolis of a developing and second most populous country in the world. However, there are certain study limitations. The vaccination status of the participants was preferably validated with the official vaccination certificate, but when unavailable, it was ascertained through recall which may have resulted in an information bias. The social desirability bias of the participants may have resulted in over-reporting of the intention towards vaccination within the unvaccinated participant groups. The study results also may lack generalizability in areas with limited vaccine availability, accessibility, and comparatively lower educational status of the residing population.

## Conclusions

In conclusion, the present study observed the lack of vaccine acceptance in nearly one in three individuals with considerable delays in second-dose vaccination. Males, older adults (>50 years), with higher educational status and a history of COVID-19 infection were more likely to be fully vaccinated with both doses compared to females, younger adults, comparatively lower educational status, and in the absence of history of COVID-19 infection. Concerns over safety and efficacy were the major drivers of vaccine hesitancy. Nevertheless, despite inhibitions and low intention to vaccinate children before the Omicron wave of the pandemic, with the subsequent availability of vaccines, most children were vaccinated by their parents. Future pandemic preparedness should seek to identify lessons from the current COVID-19 pandemic as to the health system, health policy related, and other factors that enabled population level transition from vaccine hesitancy to vaccine confidence. 
